# Meta-analysis on the effect of the N363S polymorphism of the glucocorticoid receptor gene (*GRL*) on human obesity

**DOI:** 10.1186/1471-2350-7-50

**Published:** 2006-05-25

**Authors:** Amelia Marti, M Carmen Ochoa, Almudena Sánchez-Villegas, J Alfredo Martínez, Miguel Angel Martínez-González, Johannes Hebebrand, Anke Hinney, Helmut Vedder

**Affiliations:** 1 Department of Physiology and Nutrition, University of Navarra, Pamplona, Spain; 2 Department of Preventive Medicine and Public Health, University of Navarra, Pamplona, Spain; 3 Department of Clinical Sciences, University of Las Palmas de Gran Canaria, Spain; 4 Department of Child and Adolescent Psychiatry, Rheinische Kliniken Essen, Essen, Germany; 5 Department of Psychiatry and Psychotherapy, Philipps-University of Marburg, Marburg, Germany

## Abstract

**Background:**

Since both excess glucocorticoid secretion and central obesity are clinical features of some obese patients, it is worthwhile to study a possible association of glucocorticoid receptor gene (*GRL*) variants with obesity. Previous studies have linked the N363S variant of the *GRL *gene to increased glucocorticoid effects such as higher body fat, a lower lean-body mass and a larger insulin response to dexamethasone. However, contradictory findings have been also reported about the association between this variant and obesity phenotypes. Individual studies may lack statistical power which may result in disparate results. This limitation can be overcome using meta-analytic techniques.

**Methods:**

We conducted a meta-analysis to assess the association between the N363S polymorphism of the *GRL *gene and obesity risk. In addition to published research, we included also our own unpublished data -three novel case-control studies- in the meta-analysis The new case-control studies were conducted in German and Spanish children, adolescents and adults (total number of subjects: 1,117). Genotype was assessed by PCR-RFLP (*Tsp509I*). The final formal meta-analysis included a total number of 5,909 individuals.

**Results:**

The meta-analysis revealed a higher body mass index (BMI) with an overall estimation of +0.18 kg/m^2 ^(95% CI: +0.004 to +0.35) for homo-/heterozygous carriers of the 363S allele of the *GRL *gene in comparison to non-carriers. Moreover, differences in pooled BMI were statistically significant and positive when considering one-group studies from the literature in which participants had a BMI below 27 kg/m^2 ^(+ 0.41 kg/m^2 ^[95% CI +0.17 to +0.66]), but the differences in BMI were negative when only our novel data from younger (aged under 45) and normal weight subjects were pooled together (-0.50 kg/m^2 ^[95% CI -0.84 to -0.17]). The overall risk for obesity for homo-/heterozygous carriers of the 363S allele was not statistically significant in the meta-analysis (pooled OR = 1.02; 95% CI: 0.56–1.87).

**Conclusion:**

Although certain genotypic effects could be population-specific, we conclude that there is no compelling evidence that the N363S polymorphism of the *GRL *gene is associated with either average BMI or obesity risk.

## Background

Since 1990, important efforts have been made to identify genes influencing complex traits, often by relying on the use of *a priori *selected candidate genes. According to the updated 2004 version of the Human Obesity Map, 113 candidate genes showed initial association with obesity, out of which only 18 genes are supported by at least five positive studies [1-3]. Since both excess glucocorticoid secretion and central obesity are clinical features of some obese patients, it is worthwhile to study a possible association of glucocorticoid receptor gene (*GRL*) variants with obesity [4,5]. The GRL gene is located on chromosome 5q31.3 and is also named nuclear receptor subfamily 3, group C, member 1 (NR3C1). There are three known polymorphisms in the *GRL *gene -*Bcl*I, ER22/23EK, N363S- with the last two involved an aminoacid sequence change [6-17]. The N363S variant of the GRL gene increases the trans-activating capacity both *in vivo *e *in vitro*, and it has been shown to be associated with an increased sensitivity to glucocorticoids *in vivo*. An association of the variant N363S with measures of increased glucocorticoid effects such as more body fat, a larger insulin response to dexamethasone and a less lean-body mass has been reported [5,7].

The polymorphism N363S (rs6195) in exon 2 of the *GRL *(U78506) is present with allele frequencies ranging from 0.7% in a South Asian population [13] to 27% in Australian subjects [12]. This polymorphism was reported to be associated with an increased body mass index (BMI), an increased weight gain or a higher waist to hip ratio in some populations, i. e. in Dutch, Anglo-Celtic, French and Australian individuals [6,7,11,15,16], while other studies performed on Danish, Swedish, and South Asian subjects were not able to confirm this finding [9,10,12-14,17]. The disparity of the results may partly be attributed to insufficient power in some of these studies, false positive or negative findings, or other reasons such as methodological issues, or the heterogeneity in age, sex, ethnic origin or average BMI of participants.

To further explore the role of the N363S polymorphism of the *GRL *gene in human obesity, we examined novel case-control data from two Spanish and one German study and included the results in a meta-analysis of a large number of previously published studies.

To overcome the limitations of the previous research work, we pooled data from different studies including our own results to rigorously assess any association and ascertain the likelihood and magnitude of the association between the allelic variants and the phenotype of interest [18-21]. Because non-significant results can be due to type 2 errors, meta-analyses greatly increase the power [20] and thus reduce the probability of false negative findings. On the other hand, some of the apparently significant results are simply due to type 1 errors resulting from spurious findings. Consequently, data pooling also reduces the type 1 error rate.

Therefore, we performed a meta-analysis by combining our data from three novel case-control studies (n = 1,117) with a number of previously reported studies (n = 4,792), to gain convincing evidence for (or against) the association between the N363S polymorphism of the *GRL *gene and obesity risk. A family-based study was also conducted in 124 German obesity trios (obese child or adolescent and both of their biological parents).

## Methods

### Study population

The study population was recruited from Spain (Navarra) and Germany. Two Spanish studies following a case-control design were conducted as described in detail elsewhere [22,23], see Table 1 for phenotypic details. The first case-control study comprised 370 children and adolescents (185 obese and 185 control subjects). Obesity was defined according to a body mass index (BMI) above the 97th percentile for sex and age according to Spanish reference data [22]. Controls had a BMI below the 90th percentile. The participants were mostly Caucasians (98.4%) originating from an homogeneous population of a limited geographical area in Northern Spain (Navarra) except for a small proportion of subjects (1.6%) that were non-Caucasian (3 subjects from South American, 1 Hindu, 1 Afro-American and 1 Gypsy subject). The second (adult) case-control study included 159 obese subjects with a BMI ≥ 30 kg/m^2 ^and 154 lean controls with BMIs below 25 kg/m^2 ^[23]. All participants were Caucasians originating from a homogeneous population of a limited geographical area in Northern Spain (Navarra). The German study included 178 extremely obese children and adolescents – BMI above the 99th percentile for age and sex compared to a representative German population sample [24] – and 256 lean young adults (BMI ≤ 15th percentile) serving as controls. The mean BMI (SD) was 35.6 (6.1) kg/m^2 ^for the obese group and 18.2 (1.11) kg/m^2^) for the control group. 50% of the obese subjects and 49% of the controls were male.

**Table 1:** Phenotypic characteristics of the two Spanish case-control studies

	**Navarra children**		**Navarra adults**	
	**Obese (n = 185)**	**Controls (n = 185)**	**Obese (n = 159)**	**Controls (n = 154)**

**% Male**	53%	53%	12.6%	26.6%
**Age (years)**	11.4 (11.0–11.8)	11.7 (11.3–12.1)	42 (41–44)	39 (37–40)
**BMI (kg/m^2^)**	27.6 (26.9–28.3)	19.0 (18.6–19.4)	37.6 (36.7–38.5)	22.3 (22.0–22.6)
**Percentage body fat**	34.3 (34.1–36.4)	18.2 (16.8–19.5)	43.4 (42.3–44.6)	27.5 (26.7–28.3)

Data are presented as mean (95%CI).

The German study of case-parents encompassed 124 extremely obese children and adolescents (BMI ≥ 99th BMI percentile according to German reference data [24] and both of their parents. It is worth mentioning that obese children and adolescents included in the case-parent study were independent of the individuals included in the case-control study. All participants of the German study groups were Caucasians recruited in West and South Germany.

The study was approved by the Ethics Committee of each University, and all subjects, (and, in case of minors, their parents) provided written informed consent. The reported investigation was carried out according to the principles of the Declaration of Helsinki II.

### Procedures

Extraction of genomic DNA was performed according to standard protocols. The N363S polymorphism of the *GRL *gene was analyzed by PCR-RFLP (digestion with *Tsp509I*) as described previously [11]. For validity of the genotypes, allele determination was made independently by at least two experienced individuals. Discrepancies were solved unambiguously either by reaching consensus or by repeating the experimental procedure. In addition, we checked for Mendelian inconsistencies in the trios with the program Pedcheck [25].

### Statistical analysis

Descriptive values are given as mean and standard error of the mean. Univariate statistical analysis was performed using unpaired Student t-test and Chi square test (for frequencies).

The Transmission-Disequilibrium Test (TDT) was used to assess the differential pattern of excess transmission of alleles from heterozygous parents to diseased children [26]. By sampling family trios including the affected children and adolescents (probands), the association between N363S allele and obesity would lead to a transmission type different from the expected probability of 0.5. When cases are unrelated probands, TDT represents a valid test of association, even if a population stratification is present [26].

### Meta-analysis

To systematically review differences in BMI across the presence/absence of the N363S polymorphism of the *GRL *gene (including homozygous and heterozygous subjects for the 363S allele together) we used a formal meta-analysis [27]. Procedures of formal meta-analysis have been mostly applied to combine the results from previously reported studies[28]. However, in the current meta-analysis, we also included data from three of our own studies that have not been published yet.

A Pubmed search revealed 16 studies published before April 2005 focusing on the analysis of the N363S polymorphism of the *GRL *gene in human (obese or lean) subjects. Subsequently, these were included in the present analysis. Since new information was required on the frequencies of the N363S polymorphism of the *GRL *gene according to different BMI categories, we personally contacted all authors to obtain additional information by personal communication (12 of the 16 studies). Therefore, we were able to combine data from 12 studies with a total of 4,792 subjects from the literature and data from our present investigation on 1,117 subjects. Thus, the total number of subjects in the meta-analysis was 5,909 subjects.

The meta-analysis provides a logical structure for systematically quantifying evidence and for exploring bias and diversity in research[27]. In our meta-analysis, the presence/absence of the polymorphism was considered as the exposure, whereas a change in BMI was considered as the outcome. The average difference in BMI between individuals with or without the N363S polymorphism (homo-/heterozygous carriers of the 363S allele vs. non-carriers) of the *GRL *gene was used as the outcome.

We estimated a summary difference (d_s_) as the weighted effect size [29]:

d_s _= d_i_w_i_w_i_

d_s _is the pooled estimate across studies for the difference in the BMI (kg/m^2^) in homo-/heterozygous carriers and non-carriers of 363Ser allele, w_i _is the weight assigned to each study (w_i _is the inverse of the variance for the difference in BMI found in each study) and d_i _is the BMI difference between those with or without the N363S polymorphism in each individual study. We also calculated a 95% confidence interval (CI) for the pooled difference in BMI:

95% CI = d_s _± (1.96 × SE)

where SE is the standard error of the pooled estimate [27].

A test of heterogeneity was also calculated, estimating a Q statistic, which follows a Chi-square distribution with degrees of freedom of k-1, *k *being the number of studies included in the analysis. A two-tailed *p *value < 0.05 for this statistic parameter indicates the presence of heterogeneity, which somewhat compromises the validity of the pooled estimates [30]. For the pooled odds ratio we used the DerSimonian and Laird's random effect model [31].

Because significant heterogeneity was clearly evident in the pooled difference estimates for all studies combined, we evaluated potential sources of heterogeneity by subset analysis [27]. We analysed sub-groups using the following criteria: firstly, unpublished studies which included six datasets from the present investigation considering obese and control subjects and children and adolescents *vs*. adult subjects; secondly, published studies with one population group (nine studies); thirdly, published studies with two groups – an obese and a lean subject group (three studies, six datasets). We also repeated the meta-analysis in subgroups built according to the prevalence of the mutation -homo-/heterozygous carriers- (<5%; 5–10%: >10%), according to ethnicity, and according to the gender of participants.

## Results

### Studies on Spanish and German populations

In this work, firstly we examined the N363S polymorphism of the *GRL *gene in two Spanish case-control studies. The main phenotypic characteristics of the Spanish adults and children study groups are given in Table 1. The German population was composed of 178 extremely obese children and adolescents (mean age 14, 50% boys) and 256 lean young adults (mean age 25, 50% male) as indicated in the Methods section.

The percentages of homo-/heterozygous carriers of the 363S allele of the *GRL *gene for each study group are shown in Table 2. Values ranged from 1.26% in the Spanish adult obese subjects to 8.59% in the German control group and were higher in the German than in the Spanish participants and in controls *versus *obese subjects. All Spanish carriers were heterozygous for the polymorphism N363S of the *GRL *gene. Only one homozygous carrier for the 363S allele was found among the German extremely obese children and adolescents. All study groups fulfilled Hardy-Weinberg equilibrium.

**Table 2:** Mean and difference (95% CI) in body mass index (kg/m^2^) between individuals homozygous and heterozygous carriers of the 363S allele of the *GRL *gene and homozygotes for the N363 allele in studies with two population group

	Normal weight subjects								Obese subjects							
Study	N363/363S 363S/363S		N363/N363		Mean difference	95% CI		% carriers	N363/363S 363S/363S		N363/N363		Mean difference	95% CI		% carriers
										
	n	Mean BMI	n	Mean BMI					n	Mean BMI	n	Mean BMI				

**Published data**																
Lin et al. 1999	27	26.5	171	25.7	0.8	-0.9	2.5	13.64	39	43.6	107	42.9	0.7	-2.5	3.8	26.71
Lin et al. 2003	33	26.6	230	25.6	1.0	-0.5	2.5	12.55	39	43.5	111	43.1	0.4	-2.7	3.5	26.00
Echwald et al. 2001	79	26.4	775	26.1	0.2	-0.6	1.1	9.25	66	35.9	675	35.7	0.2	-1.2	1.7	8.91

Total (n)	139		1176						144		893					

**Novel data**																
Spain (Navarra)																
Adults	7	22.4	147	22.3	0.1	-1.3	1.4	4.54	2	37.1	157	37.6	-0.5	-8.5	7.5	1.26
Children	8	18.6	177	18.9	-0.3	-2.2	1.6	4.32	4	24.3	181	27.6	-3.3	-7.9	1.3	2.16
Germany																
Children and adolescents	22	17.6	234	18.3	-0.7	-1.2	-0.2	8.59	8	41.3	170	35.4	5.9	1.7	10.2	4.49

Total (n)	37		558						14		508					

In the study by Echwald et al (2001) only men were recruited from Copenhagen, Denmark, mean age = 45. The study by Lin et al (1999) included men (53%) and women (47%), mean age = 50, from Sydney (Australia), the study by Lin et al (2003) included men (80%) and women (20%), mean age = 45, from Sydney (Australia). The Navarra adults study enrolled men (20%) and women (80%), mean age = 40, from Navarra (North of Spain). The Navarra children study encompassed boys (53%) and girls (53%), mean age = 11 from Navarra (North of Spain). The German children study consisted of boys (50%) and girls (50%), mean age = 14 from West and South of Germany.

Apart from these three study groups, a family-based association study was also conducted separately in 124 German trios. 15 parents were heterozygous for the N363S polymorphism of the *GRL *gene, of which 8 transmitted the allele 363S (*p*-value = 0.99). Hence, we detected no transmission disequilibrium for the N363S polymorphism of the *GRL *gene in the population studied.

### Published studies included in the meta-analysis

The number and mean BMI for homo-/heterozygous carriers the 363S allele of each study are described in Table 2 (studies with one obese and one normal weight subject group) and Table 3 (only one group). We combined data from 12 studies from the literature having either one or two groups of subjects (cases and controls) with a total of 4,792 subjects. As seen in Table 4, we performed several types of analysis. We pooled published studies with only one group (9 studies), unpublished studies including 6 datasets from the three studies of the present investigation, considering obese and control subjects separately; and published studies with two groups of subjects (three studies).

**Table 3:** Mean and difference (95% CI) in body mass index (kg/m^2^) between individuals homozygous and heterozygous carriers of the 363S allele of the *GRL *gene and homozygotes for the N363 allele in studies with one population group

**Study**	**363S/N363 363S/363S**		**N363/N363**		**Mean difference**			**% carriers**	**City, origin**	**Age, gender**	**Ref.**
	
	**n**	**Mean BMI**	**n**	**Mean BMI**	**95% CI**						
Huizenga 98	12	28.1	198	26.6	1.5	1.3	1.7	5.7	Rotterdam, Netherlands	66–68, both	6
Halsall 00	38	26.3	453	25.8	0.5	0.4	0.6	7.7	Isle of Ely, UK	54, NA	7
Rosmond 01	25	26.0	243	26.2	-0.2	-1.8	1.4	9.3	Gotenburg, Sweden	NA, men	8
Dobson 01	23	26.1	352	26.6	-0.5	-2.6	1.7	6.1	Newcastle, UK	54, both	9
Morris 03	46	41.7	122	45.0	-3.3	-6.3	-0.3	27.4	Sydney, Australia	NA, NA	11
Rosmond 03	15	27.5	141	27.1	0.4	-1.6	2.4	9.6	Sävedalen, Sweden	NA, men	10
Roussel 03	20	29.6	349	28.0	1.6	-0.5	3.7	5.4	Paris, France	62, both	12
Wüst 04	16	21.1	92	22.0	-0.8	-2.4	0.7	14.8	Trier, Germany	18, men	14
Syed 04	2	28.2	293	27.1	1.1	-5.3	7.4	0.7	South Asian, UK	51, both	13

**Total (n)**	**197**		**2243**								

NA: not available

**Table 4:** Pooled differences (95% CI) in body mass index (kg/m^2^) between individuals homozygous and heterozygous carriers of the 363S allele of the *GRL *gene and homozygotes for the N363 allele. Fixed effects model

**Studies**	**Difference in BMI (kg/m^2^) Pooled estimates**	95% CI	**Q statistic**	**k**	**p^#^**
Overall studies	+0.18	+0.004 to +0.35*	84.47	21	<0.0001
BMI < 27 kg/m^2^	+0.14	-0.052 to +0.32	29.15	10	0.0006
BMI > 27 kg/m^2^	+0.37	-0.047 to +0.79	54.31	11	<0.0001
Adults (>25 years)	+0.51	+0.31 to +0.72*	27.47	16	0.0252
Children and adolescents (<25 years)	-0.67	-0.99 to -0.35*	19.94	5	0.0005
- Ethnicity					
European Countries	+0.16	-0.01 to +0.34	74.84	15	<0.0001
Australia	+0.45	-0.34 to +1.24	9.03	5	0.06
- Gender					
Men	-0.37	-1.25 to +0.51	1.20	3	0.5487
Men and women	+0.20	+0.02 to +0.37	81.76	18	<0.0001
- Mutation prevalence					
(<5%)	-0.54	-0.96 to -0.12	20.69	6	0.0009
(5–10%)	+0.34	+0.15 to +0.54	37.74	9	<0.0001
(>10%)	+0.10	-0.57 to +0.77	11.84	6	0.037
One population					
BMI < 27 kg/m^2^	+0.41	+0.17 to +0.66*	5.88	4	0.1177
BMI ≥ 27 kg/m^2^	+1.19	+0.64 to +1.74*	13.34	5	0.0097
Normal weight subjects					
already published data	+0.56	-0.07 to +1.19	1.04	3	0.5941
novel data	-0.50	-0.84 to -0.17*	1.72	3	0.4227
Obese subjects					
already published data	+0.32	-0.82 to +1.46	0.07	3	0.9633
novel data	-1.26	-2.10 to -0.49*	15.77	3	<0.001

k: number of studies;

^# ^test for heterogeneity;

* p < 0.05 for the pooled difference in BMI.

When analyzing data from one-population studies (Table 4), a subgroup of four studies was generated considering only reports including participants with BMI values below 27 kg/m^2^. For this group, the pooled difference in BMI values was statistically significant (+0.41 kg/m^2^; [95% CI: +0.17 to +0.66]) for 363S homo-/heterozygous carriers of the 363S allele compared to non-carriers.

We also examined the pooled difference in BMI between homo-/heterozygous carriers and non-carriers of the 363S allele of the *GRL *gene in published articles with two population groups: a group of controls (normal weight subjects) and a group of cases (obese subjects). The pooled difference in BMI between homo-/heterozygous carriers and non-carriers of the 363 allele was not statistically significant when studies with normal weight subjects (+0.56 kg/m^2^; [95% CI: -0.072 to +1.19]) and obese subjects (+0.32 kg/m^2 ^[95% CI: -0.82 to +1.46]) were considered separately. However, the pooled difference in BMI was negative and statistically significant in homo-/heterozygous carriers of the S363 allele when only novel data for normal weight subjects were examined (-0.50 kg/m^2^; [95% CI: -0.84 to -0.17]). Opposite tendencies for differences in BMI were also found when performing the meta-analysis after stratifying by gender, prevalence of the polymorphism and considering children and adult populations, separately (Table 4).

**Figure 1:**
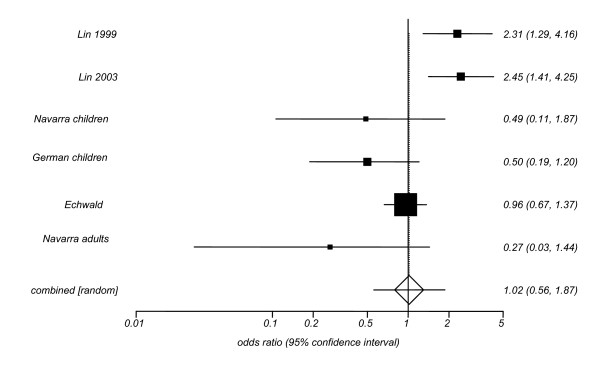
**Odds Ratios with the 95% CI for the association between presence of the N363S polymorphism of the *GRL *gene and the likelihood of being obese in the overall studies**. In the study by Echwald et al (2001) only men were recruited from Copenhagen, Denmark, mean age = 45. The study by Lin et al (1999) included men (53%) and women (47%), mean age = 50, from Sydney (Australia), the study by Lin et al (2003) included men (80%) and women (20%), mean age = 45, from Sydney (Australia). The Navarra adults study enrolled men (20%) and women (80%), mean age = 40, from Navarra (North of Spain). The Navarra children study encompassed boys (53%) and girls (53%), mean age = 11 from Navarra (North of Spain). The German children study consisted of boys (50%) and girls (50%), mean age = 14 from West and South Germany. Test for heterogeneity Q = 24.2; 5 df; p < 0.01.

**Figure 2:**
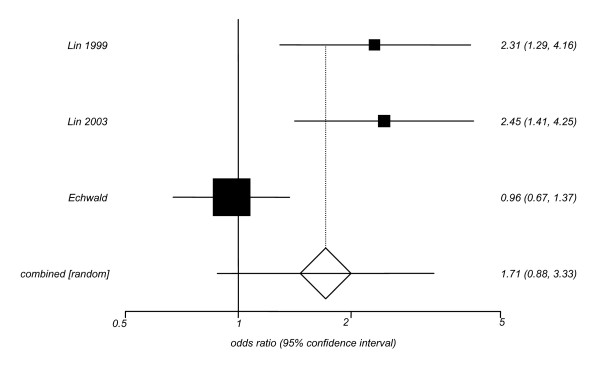
**Odds Ratios with the 95% CI for the association between presence of the N363S polymorphism of the *GRL *gene and the likelihood of being obese in the published studies**. In the study by Echwald et al (2001) only men, mean age = 45, were recruited from Copenhagen, Denmark. The study by Lin et al (1999) included men (53%) and women (47%), mean age = 50, from Sydney (Australia), the study by Lin et al (2003) included men (80%) and women (20%), mean age = 45, from Sydney (Australia). Test for heterogeneity Q = 12.3; 2 df; p < 0.01.

**Figure 3:**
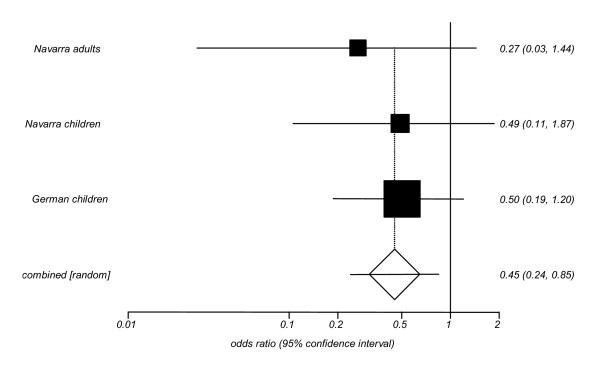
**Odds Ratios with the 95% CI for the association between presence of the N363S polymorphism of the *GRL *gene and the likelihood of being obese in the unpublished studies**. The Navarra adults study enrolled men (20%) and women (80%), mean age = 40, from Navarra (North of Spain). The Navarra children study encompassed boys (53%) and girls (53%), mean age = 11 from Navarra (North of Spain). The German children study consisted of boys (50%) and girls (50%), mean age = 14 from West and South Germany. Test for heterogeneity Q = 0.49; 2 df; p = 0.78.

We then estimated pooled odds ratios (ORs) and their 95% confidence intervals (CIs) to further explore the association between the N363S polymorphism of the *GRL *gene and the likelihood of being obese (Figures 1, 2, 3). The relative risk of obesity linked to the N363S polymorphism was not significant (pooled OR = 1.71; [95% CI: 0.88–3.33]), when data from previously published reports were used. If only the new data obtained from the present investigation were included, the OR for obesity of homo-/heterozygous carriers of the 363S allele was statistically significant, but in the opposite direction (pooled OR = 0.45; [95% CI: 0.24–0.85]). When these new data were pooled with previous findings in the meta-analysis, the overall risk of obesity linked to the polymorphism N363S was not statistically significant, revealing a pooled OR of 1.02 [95% CI: 0.56–1.87].

## Discussion

The present study was aimed to assess the effect of the N363S polymorphism of the *GRL *gene on the incidence of obesity. Although procedures of meta-analyses have mostly been applied to comprehensively examine the results from previously reported studies, we here also include novel data from three studies derived from Spanish and German populations of our group that have not been published so far. Moreover, the meta-analysis was performed only after relevant data that were not available in the original publications were obtained contacting directly with the authors. The new data set was generated using two different strategies: the first was to match controls to cases by gender and age, whereas the second one was performed as a case-parent study that can be considered as a special type of case-control study in which cases (unrelated probands) and pseudocontrols (parental non-transmitted alleles) are matched by ethnic origin. The magnitude of the association can be assessed by odds ratios (OR) in the case-control study, while the deviations of the transmission probabilities from the expected value under the null hypothesis of no-association (50%) are related to the genotype relative risk [32]. The close proximity of point estimates for ORs (Figure 3) with values below 1.0 and the transmission probabilities found in the case-parent study do not suggest a positive association between the N363S polymorphism of the *GRL *gene and childhood obesity in the German population.

We further addressed the general prevalence of the N363S polymorphism of the *GRL *gene and found it to be slightly more frequent in the German than in the Spanish study groups. Across populations, the genotype frequency of the polymorphism of the *GRL *gene varies widely. In Europe, it ranges from 4% to 6% for homo-/heterozygous carriers of the 363S allele in German, Spanish, French or British subjects and between 9% and 15% in Swedish and Danish individuals [6,10,11,14,17] Australian populations have higher frequencies of homo-/heterozygous carriers of the 363S-allele, ranging from 12–13% in normal weight individuals to 26–27% in obese subjects (Tables 2 and 3; [12,15,16]). There are also important differences in the genotype frequency of the polymorphism in obese and non-obese subjects in several other populations. Surprisingly, for the Spanish and German normal weight groups, the number of homo-/heterozygous carriers of the 363S allele of the *GRL *gene was higher compared to that found in obese subjects of the same population groups (Table 3).

To confirm the strength of our findings and because inconsistent data have been reported on the relationship between the N363S polymorphism of the *GRL *gene and obesity phenotypes [33, 34], after adding our own new data to previously published results, data from 5,909 unrelated subjects from a diverse ethnic backgrounds were pooled together for the analysis of BMI differences between homo-/heterozygous carriers and non-carriers of the 363S allele of the *GRL *gene. The overall pooled estimated genotype frequency for homo-/heterozygous carriers of the 363S allele was 4.47% (95% CI: 4.24–5.30) with a substantial heterogeneity (p < 0.001). Interestingly, for BMI differences, the overall estimation was +0.18 kg/m^2 ^(95% CI: +0.004 to +0.35) higher in homo-/heterozygous carriers of 363S allele of the *GRL *gene than in non-carriers, also with an important heterogeneity (p < 0.001). It is worthwhile to mention that the pooled analysis (i.e. the "one population studies") revealed a large range for BMI: from non-obese (mean BMI = 21.1 kg/m^2^, ref. 14) to severely-obese individuals (mean BMI = 41.7 kg/m^2^, ref 11) and also with regard to age: from 18 years [14] to 66–68 years [7]. Moreover, three studies were only conducted with men [9,10,14], while four studies included male and female subjects [6,7,11,13]. Disparities in BMI across studies as well as age and sex variability may at least partly account for the observed heterogeneity (Table 2).

Interestingly, when a subgroup of data was generated considering only reports whose participants showed a BMI below 27 kg/m^2^, the pooled difference in BMI values for homo-/heterozygous carriers of the 363S allele vs. non-carriers was statistically significant and homogeneous (+0.41 kg/m^2^; 95% CI: +0.17 to +0.66) with a *p*-value for heterogeneity of 0.12. Funnel plots of effect estimates (differences in BMI) against sample size showed symmetrical distribution suggesting that there was no apparent publication bias in the available studies.

With regard to the meta-analysis, a drawback of the OR analysis is the use of different criteria to define obese and non-obese individuals in the various studies. Some examinations (e.g. Echwald et al. 2001, and Navarra studies) were based on rather low BMI thresholds for obesity, whereas others included morbidly-obese patients with BMI values well above 35 kg/m^2^[16]. Moreover, there are important differences in age and sex among the groups analysed. Because the respective information was not provided in all studies, age and sex effects could not be adequately addressed in the current meta-analysis.

Two groups of children and adolescents (boys and girls) were included in the German and Spanish studies as well as one large group of young men with a mean age of about 27 years [17]. The other three studies were conducted in adults. In two studies [15,16], the criteria for age and percentage of males were quite similar -an age range of 48–52 years and a percentage of 49–57% of males- while in the third, the mean age of subjects was different (39–42 years old) as was the percentage of males (12–26%, Table 1).

Overall, our analyses did not indicate an increased risk for the development of obesity for homo-/heterozygous carriers of the S363 allele of the *GLR *as shown by a pooled OR of 1.02 (95% CI: 0.56–1.87). Interestingly, an inverse association (OR = 0.45) was obtained after pooling the German and Spanish studies for homo-/heterozygous carriers of the 363S allele compared to non-carriers, suggesting population differences for this parameter. Beyond the already mentioned limitations of our meta-analysis, our study provides a sound base for the further investigation on the role of the polymorphism N363S of the *GRL *gene in large population samples of diverse ethnic origins [34]. It also yields a much better opportunity for the detection of significant effects in sub-group analyses of homogeneous data. Thus, the meta-analysis represents a useful strategy to assess the potential contribution of variants with rather low allele frequencies (i.e. the N363S polymorphism of the *GRL *from 4 to 9%), which may exert only moderate effects on polygenic disorders.

## Conclusion

Although certain genotypic effects could be population-specific, we conclude that there was no compelling evidence that the N363S polymorphism of the *GRL *gene is associated with the average BMI or with an increased prevalence of obesity, in the large number of human studies included in our analyses.

## Competing interests

The author(s) declare that they have no competing interests.

## Authors' contributions

AM participated in the design of this study, recruited the meta-analysis data, contributed to the analysis and interpretation of the data, was responsible for Spanish subject enrolment and draft the manuscript. MCO carried out the laboratory analyses, participated in the analysis and interpretation of the data and helped to draft the manuscript. ASV made the statistical analysis and figures. JAM ad MAMG participated in the design of this study and in the analysis and interpretation of the data. JH, AH and HV participated in the initiation and design of this study and in the analysis and interpretation of the data and were responsible for German subjects enrollment. All authors read and approved of the final manuscript.

## Pre-publication history

The pre-publication history for this paper can be accessed here:


